# Setting scientific names at all taxonomic ranks in italics facilitates their quick recognition in scientific papers

**DOI:** 10.1186/s43008-020-00048-6

**Published:** 2020-11-17

**Authors:** Marco Thines, Takayuki Aoki, Pedro W. Crous, Kevin D. Hyde, Robert Lücking, Elaine Malosso, Tom W. May, Andrew N. Miller, Scott A. Redhead, Andrey M. Yurkov, David L. Hawksworth

**Affiliations:** 1 Department of Biological Sciences, Goethe University, Institute of Ecology Evolution and Diversity, Max-von-Laue-Str. 13, 60439 Frankfurt am Main, Germany; 2 Senckenberg Biodiversity and Climate Research Centre (BiK-F), Senckenberganlage 25, 60325 Frankfurt am Main, Germany; 3 Genetic Resources Center, National Agriculture and Food Research Organization, 2-1-2 Kannondai, Tsukuba, Ibaraki, 305-8602 Japan; 4 Westerdijk Fungal Biodiversity Institute, Uppsalalaan 8, 3584CT Utrecht, the Netherlands; 5 Center of Excellence in Fungal Research, Mae Fah Luang University, Chiang Rai, 57100 Thailand; 6 Botanischer Garten und Botanisches Museum, Freie Universität Berlin, Königin-Luise-Straße 6-8, 14195 Berlin, Germany; 7 Departamento de Micologia. Recife, Universidade Federal de Pernambuco, Centro de Biociências, Recife, 50.740-600 Brazil; 8 Royal Botanic Gardens Victoria, Melbourne, Victoria 3004 Australia; 9 Illinois Natural History Survey, University of Illinois, Champaign, IL 61820 USA; 10 Ottawa Research and Development Centre, Science and Technology Branch, Agriculture and Agri-Food Canada, 960 Carling Ave, Ottawa, ON K1A 0C6 Canada; 11 Leibniz Institute DSMZ-German Collection of Microorganisms and Cell Cultures, Inhoffenstrasse 7B, 38124 Braunschweig, Germany; 12 Department of Life Sciences, The Natural History Museum, Cromwell Road, London, SW7 5BD UK; 13 Comparative Plant and Fungal Biology, Royal Botanic Gardens, Kew, Surrey, TW9 3DS UK; 14 Jilin Agricultural University, Changchun, Jilin Province 130118 People’s Republic of China

**Keywords:** Format of names of taxa, Italics, Publication standards, Scientific names, Scientific practice

## Abstract

It is common practice in scientific journals to print genus and species names in italics. This is not only historical as species names were traditionally derived from Greek or Latin. Importantly, it also facilitates the rapid recognition of genus and species names when skimming through manuscripts. However, names above the genus level are not always italicized, except in some journals which have adopted this practice for all scientific names. Since scientific names treated under the various Codes of nomenclature are without exception treated as Latin, there is no reason why names above genus level should be handled differently, particularly as higher taxon names are becoming increasingly relevant in systematic and evolutionary studies and their italicization would aid the unambiguous recognition of formal scientific names distinguishing them from colloquial names. Several leading mycological and botanical journals have already adopted italics for names of all taxa regardless of rank over recent decades, as is the practice in the *International Code of Nomenclature for algae*, *fungi, and plants,* and we hereby recommend that this practice be taken up broadly in scientific journals and textbooks.

## BACKGROUND

The International Commission on the Taxonomy of Fungi (ICTF) is an international body devoted to its mission of promoting fungal taxonomy by facilitating the development of high scientific standards (Sigler and Hawksworth 1987; Seifert and Rossman 2010; Hawksworth 2015). The ICTF occasionally provides recommendations on publication and scientific standards related to fungal taxonomy, to promote them in the scientific community. Here, we encourage journals to adopt italics for formal scientific names at all ranks to facilitate rapid and unambiguous recognition of formal scientific names governed under nomenclatural Codes within publications, as compared to informal names such as those sometimes used to differentiate clades.

For more than a century it has been common practice in most scientific journals to italicize scientific genus and species names, since regardless of their etymology, these names are Latinised (ICNafp Art. 23; Turland et al. 2018). To highlight text in languages deviating from the main manuscript language, it was common practice to use italics, and very often, whole sentences or expressions were taken from other languages to retain their original content and meaning. This practice declined over time and in most disciplines, only a few foreign (mostly Latinized) language expressions continue to be used, such as de novo, et al., and in vitro. As these expressions are frequently employed, many journals no longer implement italics for them. Similarly, Latin-derived names that are treated as naturalized words, such as aquilegia, ascomycetes, and stramenopiles are not italicized when they are not used as formal scientific names. Thus, in a text dealing with ascomycetes, the group under study could either be addressed using the informal name ascomycetes or the formal name, the phylum *Ascomycota*. This distinction is important as formerly, the phylum *Ascomycota* was recognized at class level, as *Ascomycetes*, and so the use of italics, in combination with upper case, clearly sets apart such formal names (and their meaning) from informal ones.

Despite the fact that most publications in the biological sciences no longer italicize foreign language expressions, almost all still require authors to provide names of genera and species in italics. Apart from being a vestige of past publication practice, the main reason for this is that it is much easier to spot names of species set in italics. In addition, placing names of species in italics means that errors that occur during auto-correction of spelling, as often applied in word-processing programs, can be more readily detected and corrected. For example, a species epithet such as “*clandestina”* will be auto-corrected to ‘clandestine’ and such corrections would easily be missed during proofing if italicization was not used. As scientific taxon names are formal names under the strict rules of nomenclature Codes, they are ‘unique identifiers’ for taxa that ought to be highlighted in some way.

## CURRENT PRACTICE IN THE CODES OF NOMENCLATURE

### Algae, fungi, and plants

Scientific names at all ranks are italicized in the text of the current *International Code of Nomenclature for algae, fungi, and plants.* (ICNafp; Turland et al. 2018). This Code (and its preceding Codes and Rules) adopted italics for all scientific names covered by the Code from the earliest editions (e.g. Briquet 1935), apart from those of 1983 and 1988 which italicized names only at family rank and below. That policy was considered “rather illogical” in the next edition (Greuter et al. 1994) and in it, and all four subsequent editions to date, scientific names at all ranks covered by the Code have been placed in italics, while technical terms are not “in order to differentiate them from” scientific names. This practice has increasingly been adopted in a variety of botanical and mycological journals over the last 25 years. While the Code sets no binding standard, considering it “a matter of editorial style and tradition” it suggests editors and authors consider following this practice “in the interest of international uniformity” (Turland et al. 2018).

### Cultivated plants

A separate Code, the *International Code of Nomenclature for Cultivated Plants* (ICNCP) regulates the names of plants mostly below the species level which are recognized as cultivars, chimaeras, and some other special categories. This Code separated from the ICN in 1952, which had covered such plants up to that time, and the current edition (Brickell et al. 2016) makes only a single reference to italics; Rec. 8A recommends that the names of the special categories treated under the ICNCP should not be presented in italics to avoid confusion with taxa governed by the ICNafp.

### Prokaryotes

The nomenclature of bacterial organisms followed the provisions of the ICNafp, but there was a major issue over the acceptability of living cultures as types which led to the development of a separate Code first published in 1948 (Sneath 1986). The practice of placing scientific names at all ranks in italics has been followed through the various editions of this Code, and the deviation from this applied in the botanical Codes of 1983 and 1988 was ignored. Editors and authors are advised to “preferably indicate scientific names by a different type face, e.g. italic, or some other device to distinguish them from the rest of the text” in the *International Code of Nomenclature of Bacteria* (ICNB; Lapage and Sneath 1992). The Code has been renamed as the *International Code of Nomenclature of Prokaryotes* (ICNP; Parker et al. 2019) which repeats the advice of earlier editions recommending the use of italics for scientific names, and in the text uses them at all higher ranks up to and including class; the ICNP does not regulate names at ranks higher than class.

### Viruses

The nomenclature of viruses was first covered along with bacteria in a joint Code, the *International Code of Nomenclature of Bacteria and Viruses* (International Committee on Bacteriological Nomenclature 1958), but subsequently separated and has produced separate reports since 1971. *The International Code of Virus Classification and Nomenclature* (ICVCN; International Committee on Taxonomy of Viruses 2018) mandates the use of italics at all ranks in Rule 3.30 under the Rules of Orthography.

### Zoology

The *International Code of Zoological Nomenclature* (ICZN) traditionally regulates names in the family, genus, and species groups and excluded names above the family group, and in Rec. E.2 recommended that genus and species group names were placed in a different type, and noted “italics are usual” (Ride et al. 1985). No recommendation was made over how names at higher ranks should be presented, but most zoologists have not italicized them. There was, however, a reaction to the proposed use of italics for scientific names in the BioCode (see below) and the subsequent edition of the ICZN has the recommendation in Appendix B.6 that italics “should not be used for names of higher taxa” (International Commission on Zoological Nomenclature 1999), being now the only Code not recommending or employing italics for scientific names regardless of rank.

### PhyloCode

The PhyloCode, the *International Code of Phylogenetic Nomenclature* (Cantino and de Queiroz 2019) is a system for the regulation of formal scientific names introduced for phylogenetic clades, and is used by some systematists, including a few mycologists (Hibbett et al. 2018). Rec. 6.1A of the PhyloCode states that “In order to distinguish scientific names from other (e.g. vernacular) names, all scientific names should be italicized when they appear in print”. This applies to all possible ranks. Confusion with scientific names regulated by the organism-based Codes is avoided by the use of the prefix “clade” or the addition of “P” after the name either in squared brackets or as a superscript.

### Phytosociology

A system for giving formal scientific names to plant (including lichen) communities was developed in the first decades of the twentieth century, based largely on principles of botanical nomenclature. These names, “syntaxa” are given author citations and are now traditionally printed in italic type regardless of rank in the *International Code of Phytosociological Nomenclature* (Theurillat et al. 2020).

### BioCode

The production of a BioCode to regulate the scientific names of all organisms was a joint initiative between the International Union of Biological Sciences (IUBS) and the International Union of Microbiological Societies (IUMS) that started in 1994. An International Committee on Bionomenclature was established with official representatives of all five organism-based Codes. The BioCode aims to deal with the nomenclature of the future, but at the same time implementing lists of protected names to safeguard names it is desirable to keep from the past. A series of drafts were produced and discussed at a variety of international congresses, and the most current version was published in 2011 (Greuter et al. 2012). This includes the recommendation (Rec. 3A.1) that: “In order to denote a clear distinction between scientific names of organisms and designations in common language, scientific names of all ranks should appear in the same distinctive, and preferably italic, type”.

## ADVANTAGES OF ITALICIZATION

At the genus and species level, convergent evolution has resulted in morphotypes that appear similar, but are phylogenetically distinct, and can be members of several different genera within the same or other families. In such cases, the genus name is used as a noun, in lowercase, and non-italics. For example, the name *Fusarium* was originally applied morphologically to species with sporodochial or synnematous conidiomatal morphs, with phialides and multiseptate, falcate conidia with a distinct foot-like basal cell, borne in slime. Most species classified morphologically as *Fusarium* were discovered to represent a monophyletic group (Gräfenhan et al. 2011; O’Donnell et al. 2013). However, there are many fusarium-like morphs (lower case, non-italics) that, based on phylogenetic analyses, fall outside *Fusarium s.str.* and have been placed into other genera (Gräfenhan et al. 2011). Likewise, the name *Phoma* is attributed to species that cluster in a monophyletic clade along with the type species, *Phoma herbarum*, whereas species with a phoma-like morphology refers to coelomycetous fungi with phialidic conidiogenous cells, and hyaline, smooth, aseptate conidia. These organisms could belong to more than 30 genera, most of which cluster in *Didymellaceae*, or in other families of *Dothideomycetes* (Hou et al. 2020).

Lack of wide take-up of italicization for names of higher taxa might again be a reminiscence of past scientific publishing but may also be due to copy editors following general style guides (e.g. Ritter 2003; Council of Science Editors 2006). Until the advent of molecular phylogenetics, higher-level designations were usually fraught with uncertainty, due to deviating views regarding the importance of various characteristics. This is reflected in formal names such as *Gasteromycetes*, *Corticiaceae*, and *Zygomycetes*, which were originally defined on morphological features such as the closed sporocarps, crust-like appearance, and yoke-like fertilization structures, respectively. Due to polyphyly, some names at higher taxa that were formerly widely-used have been discarded, as is allowed under the ICNafp, as priority does not apply above the rank of family. For example, taxa previously placed in the phylum *Zygomycota* (and before that the class *Zygomycetes*) are now distributed among several phyla (that do not form a monophyletic group) and the name *Zygomycota* is no longer applied to any of them (Spatafora et al. 2017). While formal usage of higher taxon names such as *Zygomycota* has been discontinued, the names continue to be used in descriptive terms for fungi sharing certain morphological traits, such as corticioid or gasteromycetous or as common names such as gasteromycetes, zygomycetes, oomycetes, ascomycetes, and hyphomycetes. As a consequence of this, it was uncommon to italicize higher level names, no matter whether they were used in an informal or a taxonomic context, even though such distinction would be desirable to avoid confusion.

With increasingly well-resolved phylogenetic hypotheses, it has become possible to define synapomorphies for higher level groups and to redefine many names at the family level and above, e.g. in oomycetes (Beakes and Thines 2017; Buaya et al. 2020). Thus, consensus views on higher-level designations are starting to become feasible (e.g. Hibbett et al. 2007; Wijayawardene et al. 2020), stabilising the circumscription of taxa at these taxonomic ranks and promoting their use in a defined manner. As a consequence, e.g. a designation of oomycetes as the phylum *Oomycota* is now a well-defined concept, and names at family level and above are increasingly well-resolved and filled with meaning in terms of evolutionary knowledge. Also for this reason, it makes sense to now treat these taxa similar to species names and to encourage authors to italicize them, as adopted in ICNafp (Turland et al. 2018). With this practice higher-level taxa also become much easier to locate in the text of scientific articles, as exemplified here – the term oomycetes is present in this text four times, while the taxonomic term *Oomycota* is present just three times, but its occurrence is recognizable at a glance. The same applies to the use of *Fungi* (italics and upper case), referring to the monophyletic clade of the true fungi, as compared to fungi (not italics and lower case), which encompass all fungus-like organisms even if not directly related, such as the *Fungi*, the *Mycetozoa* (true slime molds) and the aforementioned *Oomycota*.

The latest revision of Chapter F of the ICNafp which concerns special provisions for *Fungi* and fungus-like organisms (May et al. 2019) supports the inclusion of the unique identifier, i.e. the formal registration number, of a name provided by a recognised repository after the name and in place of an author citation (Art. F.10.1). It is further recommended (Rec. F.10A) that this number be placed in squared brackets with the prefix “#” so that it can provide a direct and stable hyperlink to the record in the repository, such as *Index Fungorum* or *MycoBank*. The use of italics for all scientific names will flag to editors, copy-editors, and typesetters that such a hyperlink could be inserted, preferably through automated web services provided by the corresponding repositories. This would be a major benefit in providing immediate access to key basic information about the taxon. We also note that some journals are already embracing this possibility (e.g. *MycoKeys*).

## RECOMMENDATION

In view of the developments and advantages of italicization in facilitating the distinction between formal scientific names and informal ones used for higher taxa, clades or morphs (Table 1):

*· The ICTF recommends that editors of mycological journals and books instruct authors to italicize formal scientific names at all ranks; and*

*· The ICTF further recommends that editors of scientific journals and books permit this use of italics at least in mycological materials they publish, and that authors submitting papers on fungi also follow this practice.*

**Table 1:** Examples for the use of italics and roman type

Name	Notes
*Fungi*	Kingdom (monophyletic)
fungi	All organisms that are fungi in a morphological or physiological sense (polyphyletic)
*Oomycota*	Phylum
oomycetes	Any organism belonging to the *Oomycota*
*Phoma*	Genus
phoma-like	Any organism with characteristics of *Phoma*
agaric	Mushroom with umbrella-like pileus and lamellae
*Agaricus*	The genus of the button mushroom

We note and welcome that the practice of italicising scientific names at all ranks is already being adopted in a growing number of mycological and taxonomic journals. These include *IMA Fungus*, *Fungal Biology, Fungal Diversity*, *Mycotaxon, Mycosphere, Studies in Mycology, Persoonia, Fungal Systematics and Evolution*, *The Lichenologist,* and *Willdenowia*, among others. We are further pleased to note that this practise is also being adopted in recent mycological books (e.g. Crous et al. 2019; Moore et al. 2020; Samson et al. 2020).

Finally, we hope that in due course this practice will be followed in scientific journals and books relating to all organisms, including those governed by the ICZN, in accord with the practice in the ICNafp, PhyloCode, Prokaryote Code, Virus Code, Phytoscociological Code, and as commended in the BioCode as discussed above.

## Data Availability

Not applicable.

## References

[CR1] Beakes GW, Thines M, Simpson AGB, Slamovits CH (2017). *Hyphochytriomycota* and *Oomycota*. *Handbook of the Protists* (Archibald JM).

[CR2] Brickell CD, Alexander C, Cubet JJ, David JC, Hoffman MHA, Leslie AC, Malécot V, Jin X (2016). International code of nomenclature for cultivated plants (ICNCP or cultivated plant code).

[CR3] Briquet J (1935). International rules of botanical nomenclature.

[CR4] Buaya AT, Ploch S, Kraberg A, Thines M (2020). Phylogeny and cultivation of the holocarpic oomycete *Diatomophthora perforans* comb. nov., an endoparasitoid of marine diatoms. Mycological Progress.

[CR5] Cantino PD, de Queiroz K (2019). *PhyloCode; International Code of Phylogenetic Nomenclature.* Version 6.

[CR6] Council of Science Editors (2006). Scientific style and format; the CSE manual for authors, editors, and publishers.

[CR7] Crous PW, Verkeley GJM, Groenewald JZ, Houbraken J (2019). Fungal biodiversity.

[CR8] Gräfenhan T, Schroers H-J, Nirenberg HI, Seifert KA (2011). An overview of the taxonomy, phylogeny, and typification of nectriaceous fungi in *Cosmospora*, *Acremonium*, *Fusarium*, *Stibella*, and *Volutella*. Studies in Mycology.

[CR9] Greuter W, Barrie FR, Burdet HM, Chaloner WG, Demoulin V, Hawksworth DL, Jørgensen PM, Nicolson DH, Silva PC, Trehane P, McNeill J (1994). *International Code of Nomenclature for algae, fungi, and plants (Tokyo Code)*. [regnum Vegetabile no. 131].

[CR10] Greuter W, Garrity G, Hawksworth DL, Jahn R, Kirk PM, Knapp S, McNeill J, Michel E, Patterson DJ, Pyle R, Tindall BJ (2012). Draft BioCode (2011): principles and rules regulating the naming of organisms. *Bionomina***1**: 26-44; *Taxon***60**: 201-212. The Bulletin of Zoological Nomenclature.

[CR11] Hawksworth DL (2015). Proposals to clarify and enhance the naming of fungi under the international code of nomenclature for algae, fungi, and plants. IMA Fungus.

[CR12] Hibbett DS, Binder M, Bischoff JF, Blackwell M, Cannon PF (2007). A higher-level phylogenetic classification of the fungi. Mycological Research.

[CR13] Hibbett DS, Blackwell M, James TY, Spatafora JW, Taylor JW, Vilgalys R (2018). Phylogenetic taxon definitions for *Fungi, Dikarya, Ascomycota* and *Basidiomycota*. IMA Fungus.

[CR14] Hou LW, Groenewald JZ, Pfenning LH, Yarden O, Crous PW, Cai L (2020). The phoma-like dilemma, studies in mycology.

[CR15] International Commission on Zoological Nomenclature (1999). International code of zoological nomenclature.

[CR16] International Committee on Bacteriological Nomenclature (1958). International code of nomenclature of bacteria and viruses.

[CR17] International Committee on Taxonomy of Viruses (2018). The international code of virus classification and nomenclature.

[CR18] Lessel EF, Skerman VBD, Seeliger HPR, Clark WS, Lapage SP, Sneath PHA (1992). International code of nomenclature of bacteria (bacteriological code 1990 revision).

[CR19] May TW, Redhead SA, Bensch K, Hawksworth DL, Lendemer J, Lombard L, Turland NJ (2019). Chapter F of the international code of nomenclature for algae, fungi, and plants as approved by the 11th international mycological congress, San Juan, Puerto Rico, July 2018. IMA Fungus.

[CR20] Moore D, Robson GD, Trinci APJ (2020). 21^st^ century guidebook to fungi.

[CR21] O’Donnell K, Rooney AP, Proctor RH, Brown DW, McCormick SP, Ward TJ, Frandsen RJN, Lysøe E, Rehner SA, Aoki T, Robert VARG, Crous PW, Groenewald JZ, Kang S, Geiser DM (2013). Phylogenetic analyses of *RPB1* and *RPB2* support a middle cretaceous origin for a clade comprising all agriculturally and medically important fusaria. Fungal Genetics and Biology.

[CR22] Parker CT, Tindall BJ, Garrity GM (2019). International code of nomenclature of prokaryotes. Prokaryotic code (2008 Revision). International Journal of Systematic and Evolutionary Microbiology.

[CR23] Ride WDL, Sabrosky GW, Bernardi G, Melville RV (1985). International code of zoological nomenclature.

[CR24] Ritter RM (2003). The Oxford style manual.

[CR25] Samson RA, Houbraken J, Thrane JC, Fisvad JC, Andersen B (2020). Food and indoor fungi.

[CR26] Seifert KA, Rossman AY (2010). How to describe a new fungal species.

[CR27] Sigler L, Hawksworth DL (1987). International commission on the taxonomy of fungi (ICTF) code of practice for systematic mycologists. Mycopathologia.

[CR28] Sneath PHA, WDL R, Younès T (1986). Nomenclature of bacteria. Biological nomenclature today.

[CR29] Spatafora J, Aime M, Grigoriev I, Martin F, Stajich J, Blackwell M (2017). The fungal tree of life: from molecular systematics to genome-scale phylogenies. Microbiology Spectrum.

[CR30] Theurillat J-P, Willner W, Fernández F, Bültmann H, Čarni A, Gigante D, Mucina L, Weber H (2020). International code of Phytosociological nomenclature. Applied vegetation science.

[CR31] Turland NJ, Wiersema JH, Barrie FR, Greuter W, Hawksworth DL, Herednden PS, Knapp S, Kusber W-F, Li D-Z, Marhold K, May TW, McNeill J, Monro AM, Prado J, Price MJ, Smith GF (2018). *International Code of Nomenclature for algae, fungi, and plants (Shenzhen Code*). [regnum Vegetabile no. 159].

[CR32] Wijayawardene NN, Hyde KD, Al-Ani LKT, Tedersoo L, Haelewaters D (2020). Outline of fungi and fungus-like taxa. Mycosphere.

